# Hybrid heating systems optimization of residential environment to have thermal comfort conditions by numerical simulation

**DOI:** 10.1186/s40201-015-0202-2

**Published:** 2015-05-31

**Authors:** Nabi Jahantigh, Ali Keshavarz, Masoud Mirzaei

**Affiliations:** Mechanical Engineering Department, University of Zabol, Zabol, Iran; Mechanical Engineering Department, K. N. Toosi University of Technology, Tehran, Iran

**Keywords:** Virtual manikin, Thermal comfort, Radiant panels, Optimal conditions, Conventional heating

## Abstract

The aim of this study is to determine optimum hybrid heating systems parameters, such as temperature, surface area of a radiant heater and vent area to have thermal comfort conditions. DOE, Factorial design method is used to determine the optimum values for input parameters. A 3D model of a virtual standing thermal manikin with real dimensions is considered in this study. Continuity, momentum, energy, species equations for turbulent flow and physiological equation for thermal comfort are numerically solved to study heat, moisture and flow field. *K* − *ɛRNG* Model is used for turbulence modeling and *DO* method is used for radiation effects. Numerical results have a good agreement with the experimental data reported in the literature. The effect of various combinations of inlet parameters on thermal comfort is considered. According to Pareto graph, some of these combinations that have significant effect on the thermal comfort require no more energy can be used as useful tools. A better symmetrical velocity distribution around the manikin is also presented in the hybrid system.

## Introduction

The convectional air conditioning equipment used for heating and cooling environment systems consume a lot of energy. Hybrid heating systems consist of two types of heating systems, namely radiation and convection. They work together to provide an efficient thermal comfort within an environment. This type of system can supply continuous heating energy even when one of them is off. Therefore, this technique is a very promising method which can reduce the energy consumption without lowering the level of comfort conditions.

In general, the thermal comfort is affected by the air temperature, mean radiant temperature; air velocity and relative humidity are included in the environmental parameters whereas the activity level and metabolic rate are categorized as the personal ones. Hence, in the hybrid system, the radiant heater temperature, surfaces, location, the air flow field, and metabolism are the most important factors affecting the level of thermal comfort in an environment [1].

So far, researchers have proposed different models to obtain higher level of thermal comfort in an environment [2, 3]. Thermodynamic analysis of heat and mass of human body and its effects on thermal comfort were studied by Prek [4]. Jeet at el. [5] have evaluated the effect of high-temperature radiant heaters and windows on the thermal comfort. Despite Jeet’s study, Myhern and Holmberg [6] have shown that low radiant panel temperature is more suitable for indoor environment. A combination of flow field around a human body was studied numerically by Murakumi, at el. [7]. A more complex model which includes the real body shape and physiology was considered by Kilic and Sevilgen [8]. They evaluated the heat transfer, mass, air flow and moisture around the human model.

As of now, the effects of flow and geometric parameters on thermal comfort have been studied in several papers individually. However, no detailed combinations of their interaction effects are seen in the literature. Therefore, in this study, the effects of input parameter such as temperature, surface area, and position of the radiant heaters along with their interactions on a hybrid heating system have been investigated. In addition, the air flow parameters of the convection system were also considered. The main objective of this study is to achieve thermal comfort in an indoor environment. In this simulation, a 3D model of a virtual standing thermal manikin with real dimensions is used. A numerical method was used to solve the flow field around the manikin. Also an optimization toward thermal comfort conditions is done.

### Governing equations and solutions

The governing equations, consisting of continuity, momentum, energy, and mass transfer in a room which includes a manikin, are solved here. The radiation and turbulence equations are also considered in this problem. In addition, the Factorial Design Method (FDM) is used to analyze and optimize the variable effective parameters.

The conservation of mass can be written as [9]:

1 $$ \nabla .\left(\rho \overrightarrow{V}\right)=0 $$

The conservation of momentum is described by [10].

2 $$ \nabla .\left(\rho .\overrightarrow{V}.\overrightarrow{V}\right)=-\nabla p+\nabla .\left(\overline{\overline{\tau}}\right)+\rho \overrightarrow{g} $$

The stress tensor $\overline{\tau}$ is given by;

3 $$ \overline{\overline{\tau}}=\mu \left[\left(\nabla \overrightarrow{V}+\nabla {\overrightarrow{V}}^T\right)\right]-\frac{2}{3}\nabla .\overrightarrow{V}I $$

The energy equation is:

4 $$ \begin{array}{l}\nabla .\left(\overrightarrow{V}\left(\rho E+p\right)\right)=\nabla .\Big({k}_{eff}\nabla T-\\ {}{\displaystyle {\sum}_j{h}_j}{\overrightarrow{J}}_j+\left(\overline{\overline{\tau}}.\overrightarrow{V}\right)\Big)\end{array} $$

In Equation 4,

5 $$ E=h-\frac{p}{\rho }+\frac{V^2}{2} $$

Where *h* is defined as

6 $$ h={\displaystyle \sum_j{Y}_j}{h}_j+\frac{p}{\rho } $$

Where, *Y*_*j*_ is the mass fraction of species j and

7 $$ {h}_j={\displaystyle \underset{T_{ref}}{\overset{T}{\int }}{C}_{p,j}}dT $$

Here *T*_*ref*_ is 298.15 K.

The conservation equation for the species transport in the general form is:

8 $$ \frac{\partial }{\partial t}\left(\rho {Y}_j\right)+\nabla .\left(\overrightarrow{V}{Y}_j\right)=\nabla .{\overrightarrow{J}}_j $$

The mass diffusion ${\overrightarrow{J}}_j$ in turbulent flow is:

9 $$ {\overrightarrow{J}}_j=-\left(\rho {D}_{j,m}+\frac{\mu_t}{s{c}_t}\right)\nabla {Y}_j-{D}_{T,j}\frac{\nabla T}{T} $$

Where, *Sc*_*t*_ is the turbulent Schmidt number.

The Predicted Mean Vote, PMV which is the response of large groups of people to the temperature sensitivity of an environment based on ASHRAE Standard is stated as the following equation [11].

10 $$ \begin{array}{l}PMV=\Big(0.303 \exp \left(-0.035M\right)+\\ {}0.028\Big)L\end{array} $$

The heat balance equation of a human body is given as;

11 $$ \begin{array}{l}S=M\mp W\mp R\mp C\mp K-{E}_{sk}\\ {}-\left({C}_{res}+{E}_{res}\right)\end{array} $$

By solving the flow field, all terms in equation 11can be calculated.

When *S* = 0, the thermal equilibrium will be satisfied [6]. In equation 11 *E*_*sk*_, *C*_*res*_ and *E*_*res*_ terms can be calculated as follows

12 $$ {E}_{sk}=\frac{w\left(P-{P}_a\right)}{R_{cl}} $$

13 $$ {C}_{res}= 0.0014M\left( 34-T\right) $$

14 $$ {E}_{res}=1.72\times {10}^{-5}M\left(5867-{P}_a\right) $$

The Factorial Design Method (2^*k*^) or two level design is used here for optimization [12]. The input and output parameters’ range must also be specified. The input parameters are the area of convection flow, inlet flow rate, temperature, location, and the surface area of radiant heater. The output effective parameters are the radiation flux, convection flux, and PMV index. In this technique, the impact of the factor is defined as the difference between the high and low level of responses.

The values of input parameters based on some primary calculations are given in Table 1. The critical and major effects of parameters are defined in Pareto and normal probability graphs. Their effects can also be stated as a regression equation. Then, the model is optimized for the objective function with the method of least squares and logistic regression [13].

**Table 1:** Range of input parameters

Effect Parameter	Min.	Max.
Inlet area of the convection flow(m)	0.025	1
Inlet flow velocity(m/s)	0.05	1
heater temperature( °C)	30	90
The position of heater along vertical direction (m)	0.0	3
The position of heater along Horizontal direction(m)	0.0	2
The heater area(m^2^)	1	3.1

A 3D computational code was used to solve the flow field, energy, and mass equation. The PISO algorithm has been used [14]. In this approach, higher-order accuracy is achieved at cell faces. Pressure discretization is done by second order method. The *K* − *εRNG* is also suitable for low-Reynolds numbers and more accurate and reliable for a wider range of indoor environments and flows with heat transfer.

The *DO* radiation model is used to consider the radiation effects of heating surfaces and sources in the flow fluid simulation. It allows the solution of radiation at semi-transparent walls.

### Geometry of the problem

**Fig. 1:**
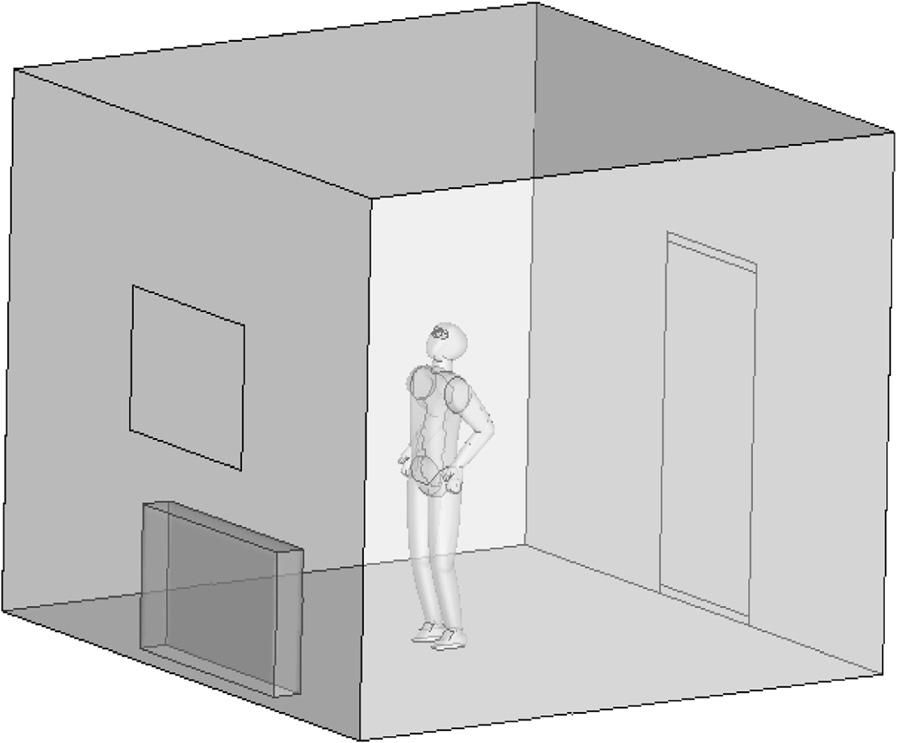
The room with heater and manikin

As be shown in Fig. 1 the problem consists of a manikin model stand and a radiant heater located within a room. One window, a door, and two vents at the bottom and top of the door have been considered for the circulation of air flow. Room dimensions are 3 × 4 × 4 m. The manikin has a height of 1.75 m and weight of 75 kg. The dimension of the window and door is 1 × 1.2 m and 1 × 2 m, respectively. The size of the inlet, outlet and the heater are assumed to be variable for the optimization purpose.

**Fig. 2:**
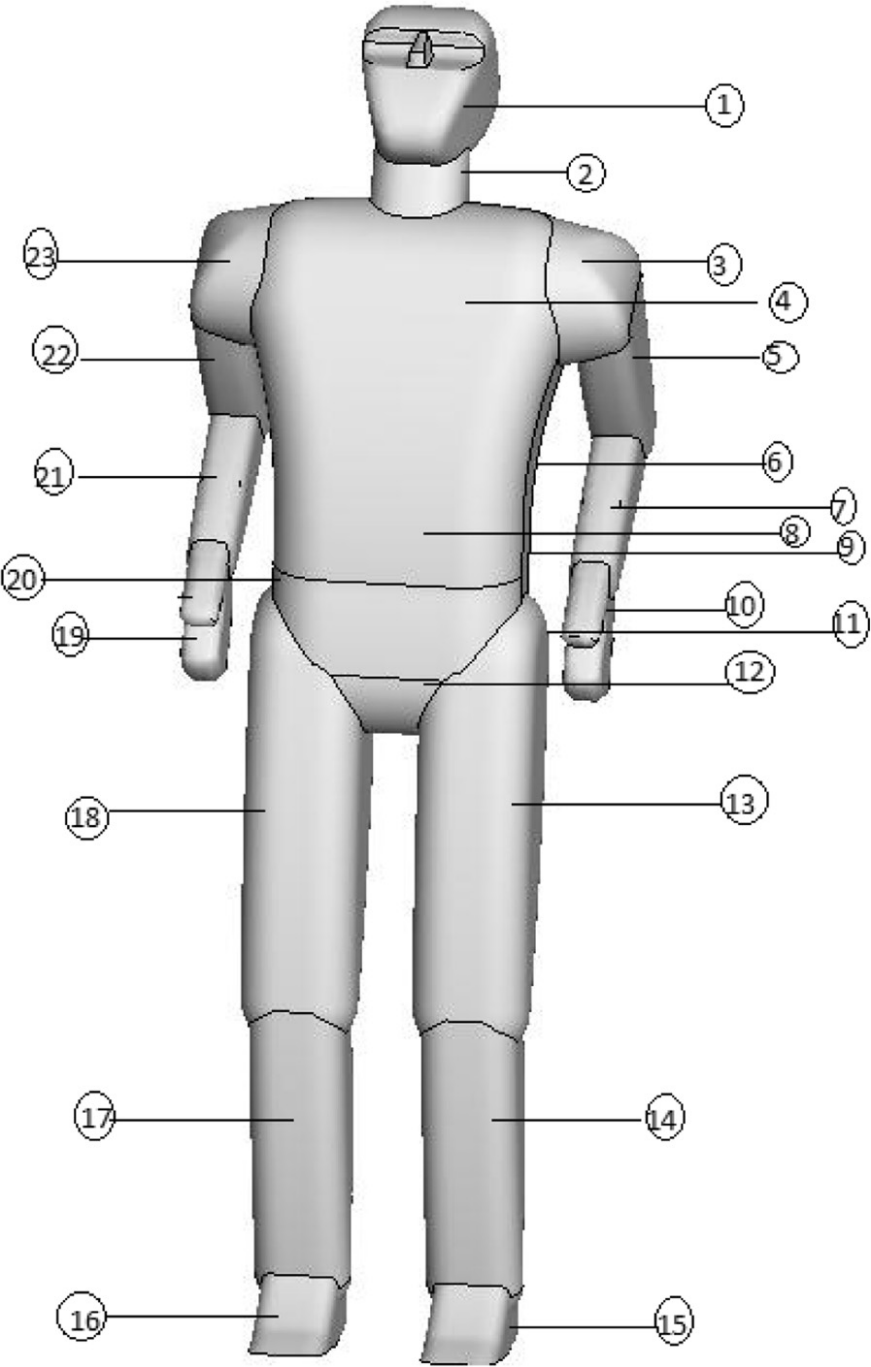
The segment of the manikin model

The segments of the manikin model have been shown schematically in Fig. 2 and their values are tabulated in Table 2.

**Table 2:** Segment of the manikin and their area

Number	Surface Name	Surface Area(m^2^)
1	Head	0.15898
2	Neck	0.028926
3,23	Right & Left shoulder	0.048249
4	Brisket	0.234812
5,22	Right & Left arm	0.098026
6,20	Right & Left	0.021141
7,21	Right & Left forearm	0.068715
8	Bowl	0.038951
9	Waist	0.210502
10,19	Right & Left hand	0.052163
11	Basin	0.041288
12	Pelvis	0.027313
13,18	Right & Left tigh	0.287548
14,17	Right & Left leg	0.160346
15,16	Right & Left foot	0.051366

This problem was first modeled using Solid Work and, then, meshed by Hypermesh software. The problem is solved for several different heater locations to optimize its position in the room.

### Computational grid

**Fig. 3:**
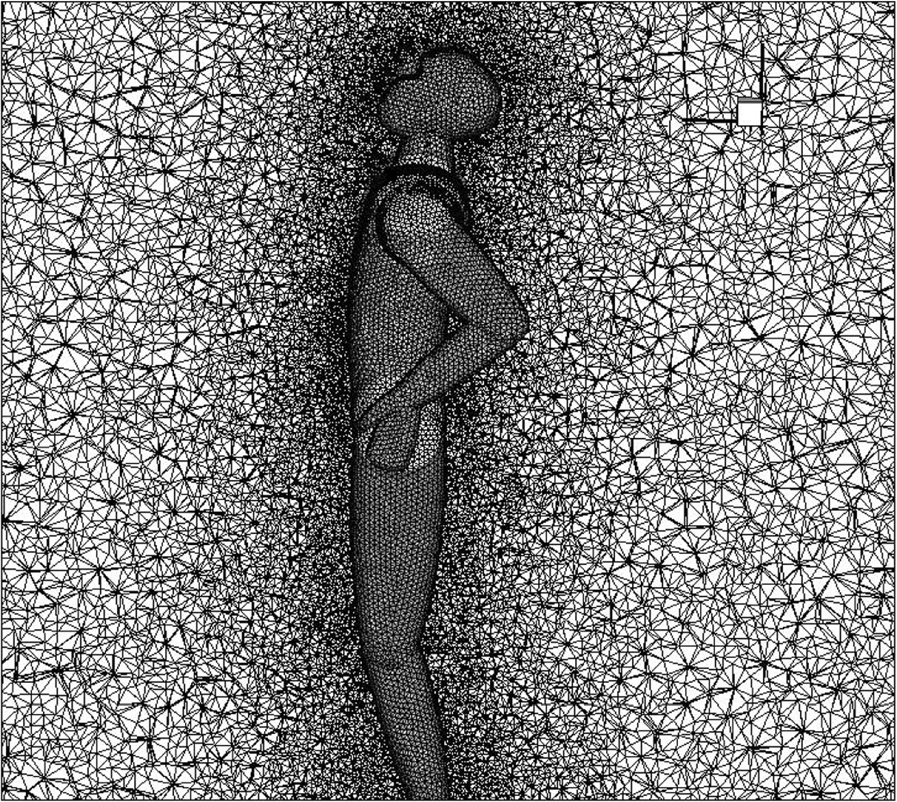
The computational domain in the room

**Fig. 4:**
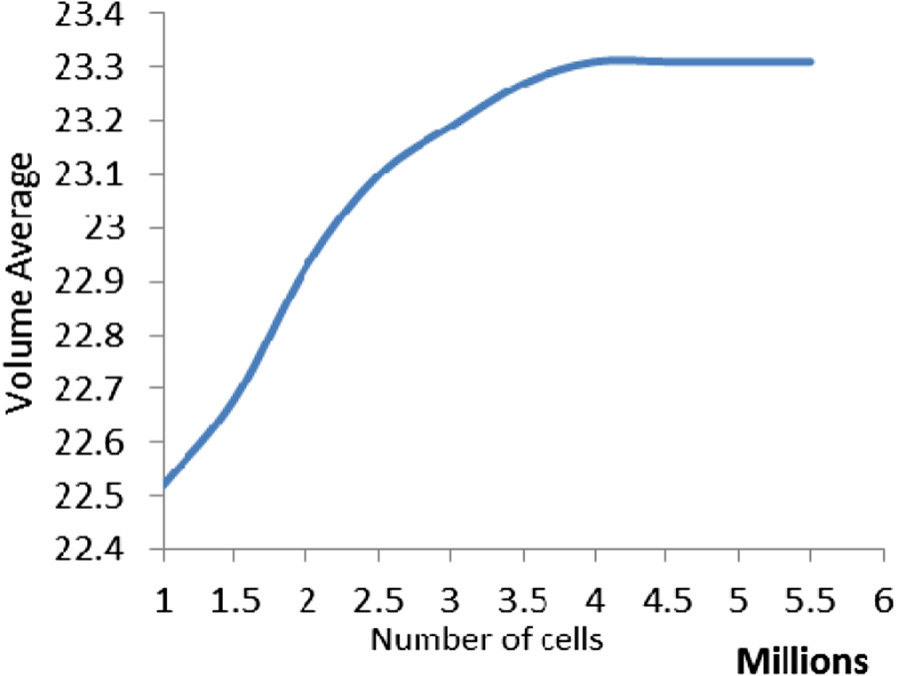
The effects of the cell number on the volume-averaged temperature of room

Due to complex geometry and fine surfaces in this problem, generating structured grid for all sub regions is not an easy task. Therefore, a combination of unstructured-structured grid system is applied using Algorithm T-Grid. It has been shown in Fig. 3. Since the flow field around the manikin includes high gradients of flow parameters, an appropriate distribution function starts from the lower layer around the surface and continues to the maximum step. The problem was solved for several different grid sizes for its independency. As shown in Fig. 4 the final results are independent numbers of grids when it is selected as 3,900,000 [15].

### Boundary conditions

The boundary conditions are given in Table 3. These values are taken based on ASHRAE standard [15]. The characteristics of building materials are given in Table 4.

**Table 3:** Boundary condition

zone	T ^∘^ *C*	type	V m/s	Mass flow
inlet	10	velocity	0.15	9.5 *gH*_2_ *O*/*kgAir*
Out let	Pressure outlet			
body	33.1	covered		10 *gH*_2_ *O*/*kgAir*
	33.7	uncovered		
wall	25	No slip	0

**Table 4:** Physical characteristic of material [15]

	Door	Wall	Window
width	50	250	25
ρ(*kg*/*m* ^3^)	700	1940	840
*C*_*p*_(*kj*/*kg.K*)	2.31	0.84	0.84
*K*(*w*/*m* ^2^.*K*)	0.173	0.06	0.2
ε	0.9	0.95	0.8

The emissivity coefficient value for the manikin has been considered as 0.98

### Validation of results

**Fig. 5:**
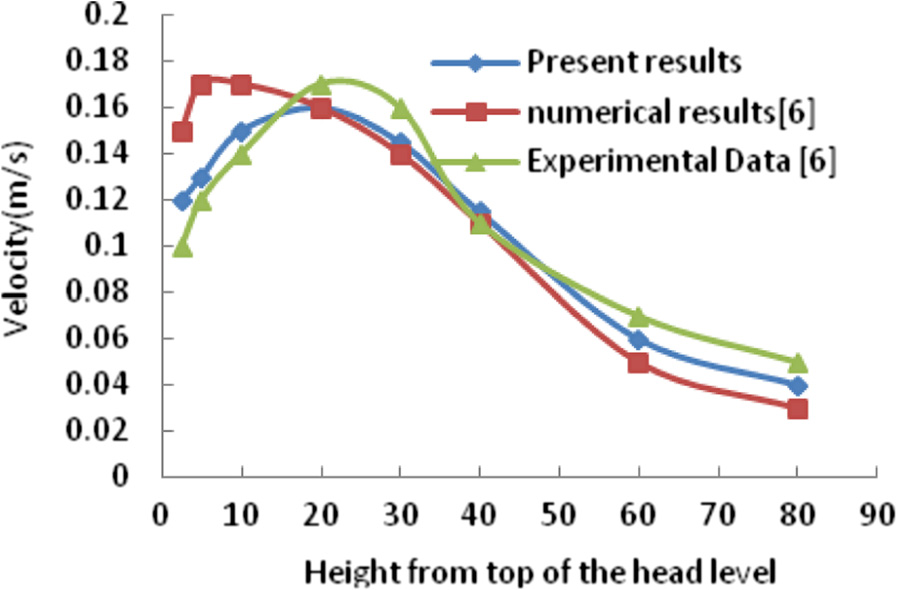
Comparison the computed velocity with Numerical and experimental results of ref. [16]

**Fig. 6:**
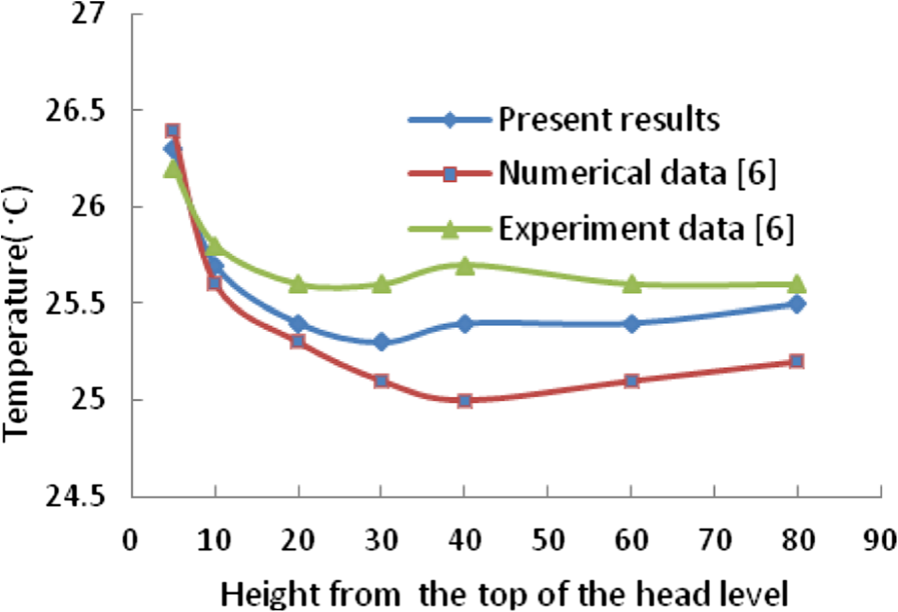
Comparison the computed temperature with Numerical and experimental results of ref. [16]

**Fig. 7:**
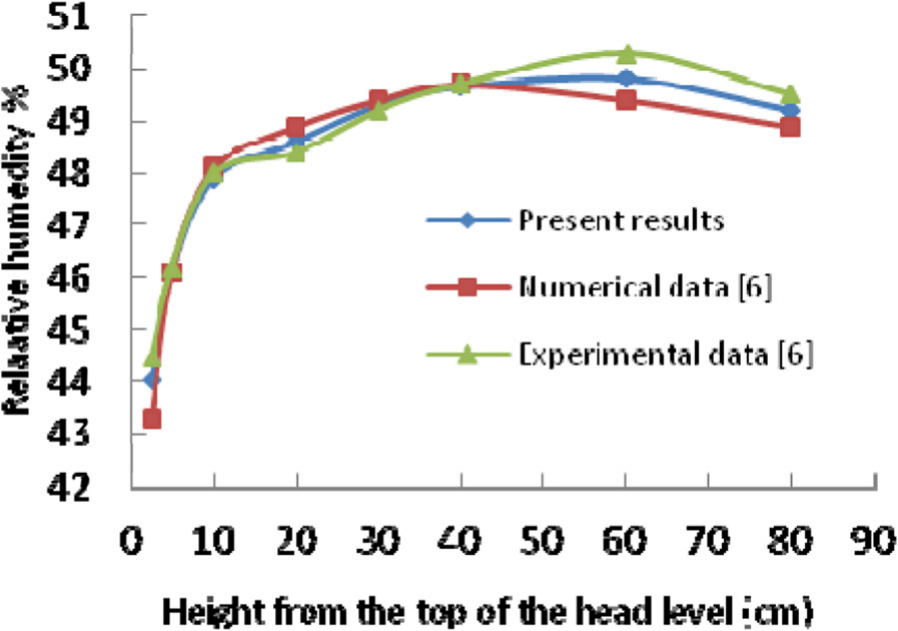
Comparison the computed RH% with Numerical and experimental results of ref. [16]

For the validation of the present model, the calculated result was compared to the available experimental data in the literature [16]. The compression for the velocity, temperature and relative humidity are shown in Figs. 5, 6 and 7, respectively. As shown in these figures, the obtained values have a high agreement with the experimental data [16]. The maximum difference between the experimental and numerical velocity, temperature and relative humidity is 0.03 m/s, 0.4 °C and 0.5 %, respectively.

## Results and discussion

### The impact of input parameters on the objective functions

**Fig. 8:**
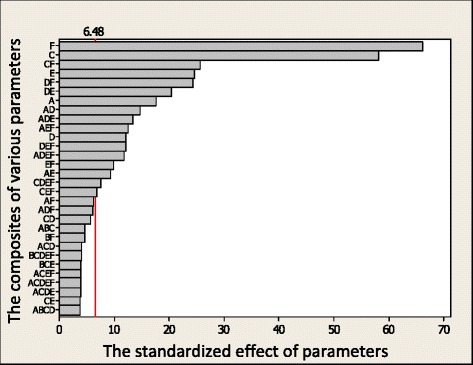
The effect of the input parameter on the PMV index

**Fig. 9:**
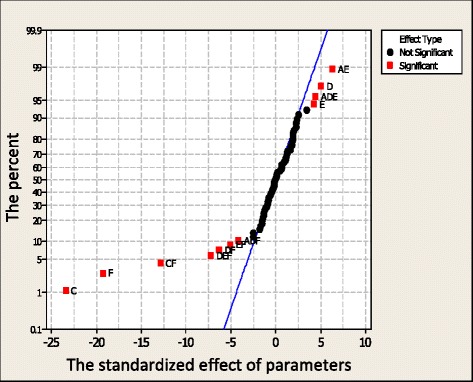
The effect of the input parameter on the radiation flux

The effect of input parameters on the objective parameters using Pareto and normal charts are compared and discussed in Figs. 8 and 9 The Pareto chart presents the significant effect of the parameters whereas the normal chart indicates their percentage. The effect of input parameters on the total heat flux, PMV index, radiation heat flux, and relative humidity are shown in these Figures, respectively.

In these Figures, the letter A is the cross section area of the inlet vent, B is the input velocity, C is the heater temperature, D and E are the positions of heaters along the horizontal and vertical directions, and F is the surface area of the heater. The impacts of these parameters as well as their combinations are studied individually.

Hence, by glancing at all Figs. 8 and 9 it can be concluded that:F has the most effect on the object function and requires more energy C has the most effect on the objective function and needs no more energy. The combination of input parameters has different impacts on the objective function in comparison to their individual ones due to their interaction. Some of these combinations which can be used as useful tools have significant effect on thermal comfort and require no more energy

### The optimization

The effect of input parameters on the objective parameters is determined in previous section and they are optimized here for different cases. In this case, the problem is optimized for the values of total flux 110w/m^2^, radiation flux 40w/m^2^, relative humidity 50 % and PMV 0.001. The optimized values are calculated and tabulated in Table 5.

**Table 5:** Optimized value for objective parameter

Input parameter	Min.	Max.	Suggested value	Optimized value	Objective parameter	Target value	Computed value along the target
Inlet velocity(m/s)	0.025	1	0.2	0.1	PMV	0.001	−0.1
Inlet area(m^2^)	0.05	1	0.4	0.12	Total flux	110	107.679
Radiation heater temperature( °C)	30	90	45	30.034	Radiation flux	40	39.778
The vertical position of heater (m)	0.0	3	.1	0.2	Relative humidity	50.	50.001
The horizontal position of heater (m)	0.0	2	2	1.85			
The heater area(m^2^)	1.0	3.1	1.5	1.749			

Locating the heater opposite the person and under the window seems to be the best achievement for the above case. In this case, minimum heat losses are obtained while having optimum PMV value

### Velocity, temperature and relative humidity distribution around the manikin

**Fig. 10:**
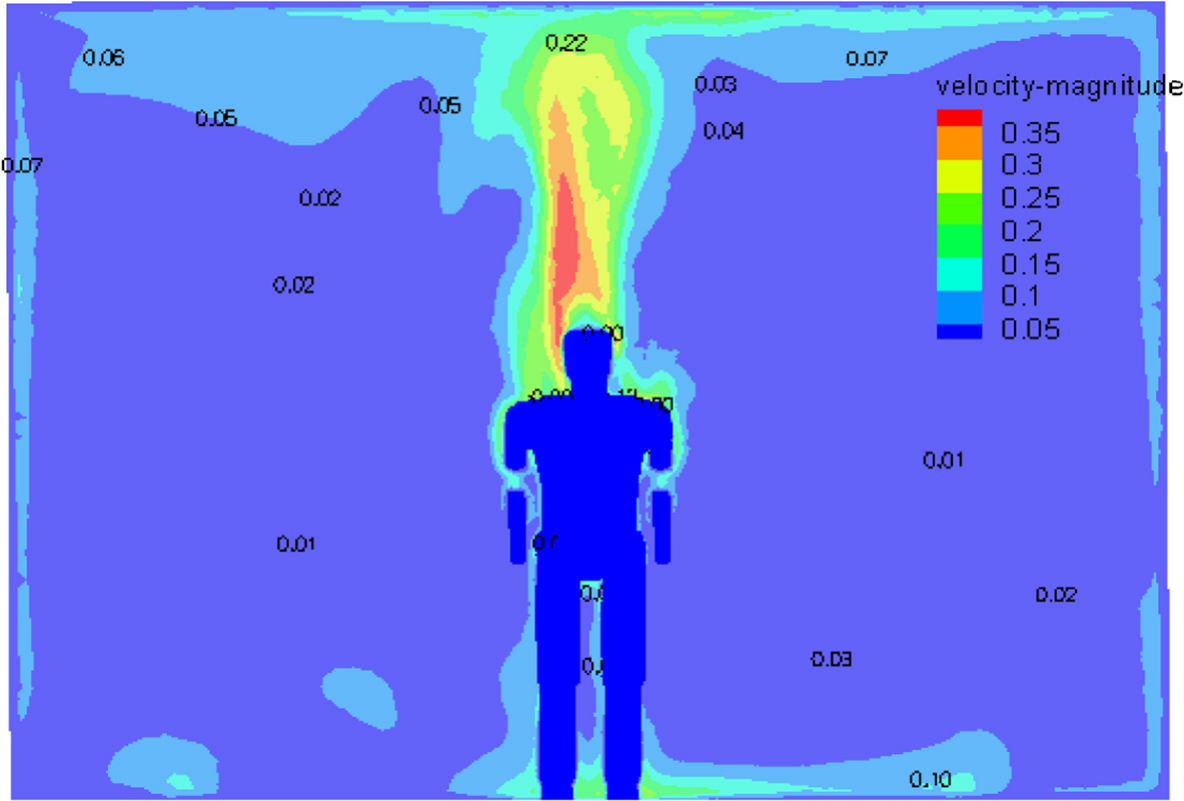
The velocity contribution for optimum case

**Fig. 11:**
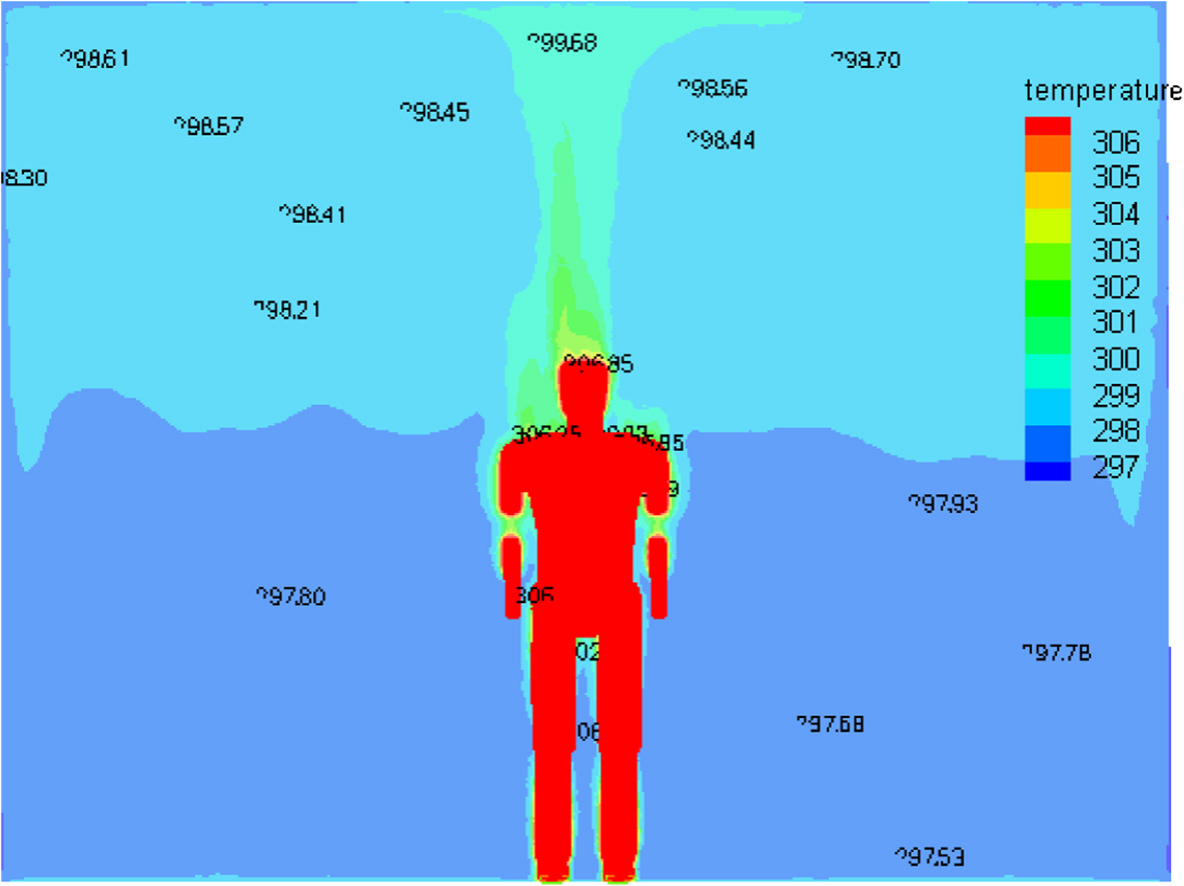
The temperature contribution for optimum case

**Fig. 12:**
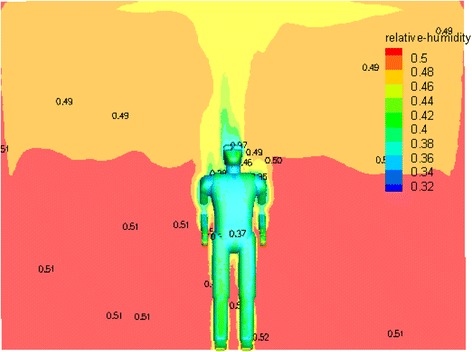
The humidity contribution for optimum case

The Velocity, temperature, and humidity contours around the manikin surface are shown in Figs. 10, 11 and 12. A better symmetrical velocity distribution around the manikin is also presented in the hybrid system. The maximum velocity occurs near the head of the manikin due to larger temperature gradient. The all distribution values around the manikin are within the ASHRAE standard [15] and there is almost no stagnation zone which provides a proper thermal comfort for a person According to temperature contour in Fig. 11.

### Thermal comfort

The fraction of convection and radiation heat flux in heating systems, convection and hybrid, for all segments of the manikin are tabulated in Table 6.

**Table 6:** Heat losses rate from body

Surface	Case-I		Case- II	
	Con. Flux(w/m^2^)	Rad. Flux(w/m^2^)	Conv. Flux(w/m^2^)	Rad. Flux(w/m^2^)
Head	43.14	38.61	39.65	41.53
Brisket	35.82	37.44	34.45	45.37
Bowl	33.73	35.90	31.92	46.03
Pelvis	31.73	32.27	30.68	39.85
Waist	33.32	31.76	32.57	38.32
Basin	33.84	34.79	31.91	40.23
Arm	37.1	32.47	36.54	41.39
Forearm	38.35	30.66	37.61	39.69
Shoulder	35.27	38.71	34.29	54.28
Thigh	34.44	31.69	33.73	48.37
Leg	41.34	36.10	40.69	43.84
Neck	33.36	35.23	38.62	45.78
foot	46.81	34.69	45.31	42.64
hand	41.69	29.24	40.15	38.69
Average	37.1	33.7	35.68	44.35

Table 7 presents the heat losses from the body and walls, and PMV values for both heating systems. As the values of PMV indicate, the heating hybrid systems can provide faster and better human thermal comfort in a residential room. The PMV value for the convection heating systems is −0.86 whereas for the hybrid system, it is 0.0034 which is almost near the ideal condition (PMV = 0) Despite the fact that the maximum velocity in this system is lower than the conventional heating system but it falls within the ASHRAE standard. This lower velocity makes lower heat losses through the walls and the manikin segments up to 25 %.

**Table 7:** Heat losses rate from body, room and PMV values in two cases

	Case 1	Case 2
T air	24.5	25.52
TMRT	24.03	25.23
Relative humidity	55.36	50.46
Convection flux	36.68	32.86
Radiation flux	33.61	45.23
losses	35.27	22.21
Total flux of the body	105.56	100.3
PMV	−0.89	0.0034
Total flux of the room	684.61	512.83

## Conclusion

In this study, two heating systems, conventional and hybrid, are individually optimized and then compared with each other to have thermal comfort conditions. The Factorial Design Method (2^K^) or two level designs has been used for optimization. The effect of input parameters and their interaction is investigated on the objective function, such as PMV index, relative humidity and heat fluxes. The effects of the input parameters in normal and Pareto charts are presented. Based on the present study, the individual and the interaction of input parameters effect are significant and different. Heater temperature has the most effect on the object function and requires more energy whereas C has the most effect on the objective function but needs no more energy thus some combination can be used as useful tools in designing heating systems. The thermal comfort can be obtained using less input energy by just increasing the surface area of the heater and its location. A better symmetrical velocity distribution around the manikin is also presented in the hybrid system. The lower velocity makes lower heat losses through the walls and the manikin segments up to 25 %. The heating hybrid systems can provide faster and better human thermal comfort in a residential room.

### Thanksgiving

I would like to thank all those who have helped me in doing the research. Also, I want to thank you for giving us the opportunity to publish our paper in the Journal of Environmental Health Science and Engineering.

## Nomenclature

*C*, Convective heat loss(w/m2)

*C*_*res*_, Convection heat loss due to respiration(w/m2)

*D*_*i,m*_, Mass diffusion coefficient for species

*D*_*T*_, Turbulent diffusivity

*E*_*res*_, Evaporative heat loss due to respiration(w/m2)

*E*, Total energy (J)

*E*_*sk*_, Evaporative heat loss of skin

$$ \overrightarrow{g} $$, Gravitational acceleration

*h*, Sensible enthalpy

*I*, Unit tensor

${\overrightarrow{J}}_j$, Diffusion flux of species

*k*_*eff*_, Effective conductivity

*k*_*t*_, Turbulent thermal conductivity

*L*, The heat load of the body

*M*, Metabolic heat production(w/m^2^)

*P*_α_, ambient pressure air (kpa)

*PMW*, Predict mean vote

*R*_*cl*_, Resistance of clothes

*R*, Radiative heat loss(w/m^2^)

*S*, Heat storage(w\m^2^)

$$ \overrightarrow{V} $$, Velocity vector

*W*, External work(w/m^2^)

*Y*_*j*_, Mass fraction of species

*μ*, Molecular viscosity

*ρ*, Density

$$ \overline{\overline{\tau}} $$, Stress tensor
